# Maternal and Neonatal Effects of Vasopressors Used for Treating Hypotension after Spinal Anesthesia for Caesarean Section: A Randomized Controlled Study

**DOI:** 10.3889/oamjms.2016.003

**Published:** 2015-12-25

**Authors:** Alma Soxhuku-Isufi, Vjollca Shpata, Hektor Sula

**Affiliations:** 1 *University Hospital of Obstetrics and Gynecology “Koço Gliozheni”, Tirana, Albania*; 2 *Faculty of Technical Medical Sciences, University of Medicine in Tirana, Tirana, Albania*; 3 *Faculty Medicine, University of Medicine in Tirana, Tirana, Albania*

**Keywords:** ephedrine, caesarean section, hypotension, maternal, neonatal outcome, phenylephrine

## Abstract

**AIM:**

The aim of the study was to examine whether ephedrine and phenylephrine were different in their efficacy for managing maternal hypotension and their effect of adverse maternal and neonatal outcome.

**METHODS:**

A double-blind randomized controlled study in healthy pregnant women ASA physical status 2, which underwent elective caesarian delivery under spinal anesthesia. Patients were randomized to receive an intravenous bolus of either phenylephrine (Ph group) or ephedrine (E group) immediately after the episode of hypotension after spinal anesthesia. Maternal and neonatal outcomes were recorded.

**RESULTS:**

Two hundred and two (202) pregnant women at term were entered in this study. There were no differences between group E and group Ph regarding the incidence of hypotension after vasopressor therapy, and the incidence of nausea and vomiting. There was no significant difference between groups in the first-minute and the 5th minute Apgar score, none of the neonates had the true fetal acidosis.

**CONCLUSIONS:**

Ephedrine and phenylephrine have the same efficacy in treating hypotension after spinal anesthesia for caesarean section. The use of Phenylephrine was associated with better fetal acid-base status, and there were no differences on Apgar score values and on the incidence of maternal bradycardia and hypotension.

## Introduction

Hypotension associated with spinal anesthesia is a common complication during caesarean section and can result in adverse effects for both mother and infant [1, 2]. When maternal hypotension associated with spinal anesthesia for cesarean section is severe and sustained, it can lead to maternal complications (nausea, dizziness, faintness) as well as impairment of the uterine and intervillous blood flow, with consecutive fetal hypoxia, acidosis, and neonatal depression [3]. The sympathectomy resulting from the neuraxial blockade is exaggerated by the physiological changes of pregnancy and puerperium, leading to hypotension in as much as 55%-90% of the mothers receiving spinal anesthesia for Cesarean section [4]. Phenylephrine and ephedrine are the vasoconstrictor agents which are currently being recommended and used for controlling hypotension, but still nowadays the choice of vasopressor has been debated [5].

Phenylephrine is the α-agonist recommended to treat hypotension that affects approximately half of caesarean sections under spinal anaesthesia [6, 7]. Also, ephedrine was the vasoconstrictor agent of choice in obstetric anaesthesia for many years due to its favourable pharmacodynamic profile; many animal models have demonstrated a marked increase in uteroplacentary blood flow [5, 8]. Results of several trials suggest that phenylephrine may have similar efficacy to ephedrine for preventing and treating hypotension during spinal anesthesia [9-12]. However, the relative effects of these vasopressors on neonatal outcome [8] and maternal outcome are unclear, and there is need for a large double-blind randomized controlled trial with emphasis on important maternal and neonatal outcomes [13].

The aim of the study was to examine whether ephedrine and phenylephrine were different in their efficacy for managing maternal hypotension after spinal anesthesia for Caesarean section and their effect of adverse maternal and neonatal outcome.

## Materials and Methods

This study was approved by the Ethics Committee of the University of Medicine, Tirana, Albania. It has been performed in accordance with the ethical standards displayed in the 1964 Declaration of Helsinki and its later amendments. Written informed consent was obtained from all patients. Data were made anonymous for analysis.

A double-blind randomized controlled study in healthy pregnant women ASA (American Society of Anesthesiologists) physical status 2, that underwent elective caesarian section under spinal anesthesia, during January 2013 - May 2015 period in University Hospital of Obstetrics and Gynecology “Koço Gliozheni” in Tirana.

The exclusion criteria were: emergency cesarean section, active labor, high risk pregnancies (multiple gestations, intrauterine growth retardation, preeclampsia maternal cardiovascular or pulmonary diseases) and other active medical disorders requiring regular medication), and any contraindication of spinal anesthesia (patient refusal, coagulopathy, hemorrhage or hypovolemic shock).

All patients fasted for at least 8 hours before induction of spinal anesthesia. Upon arrival to the operating room, all patients were monitored for basal vital signs (heart rate: HR, systolic and diastolic blood pressures: BPs, and pulse oximetry: SaO_2_). Baseline systolic arterial blood pressure was measured by averaging 3 readings taken 1 minute apart using an automated device for non-invasive blood pressure assessment. A 16-G IV catheter was placed in a peripheral vein in the patient’s upper limb, and before performing spinal anesthesia, all patients received a preload of 500 ml and a coload of 1000 ml lactated Ringer’s solution.

After completion of fluid infusions all patients received spinal anesthesia by an anesthesiologist, in sitting position at L3-L4 inter vertebral space, using 26-gauge, pencil point needle. Hyperbaric bupivacaine 12.5 mg mixed with preservative-free fentanyl 10 μg and morphine 200 μg was injected over 30 seconds. Immediately after spinal anesthesia, all patients were positioned in the supine position with left uterine displacement. Concomitantly to the intrathecal injection the patient received 10 mL/kg of lactated Ringer’s solution.

BP was controlled every minute until delivery and then every five minutes throughout anesthesia. HR and SaO_2_ were monitored throughout anesthesia.

Hypotension was considered a decrease in systolic blood pressure > 20% of baseline (prior to drugs being placed in the neuraxis) [11]. Patients were randomized to receive an intravenous bolus of either phenylephrine or ephedrine immediately after the episode of hypotension after spinal anesthesia. Randomization was performed using a computer-generated random number table. The patient and the attending anesthesiologist were blinded to the group allocation. Group allocations were placed in opaque, sealed envelopes on initial randomization. There were two groups of 101 patients: group Ph (Phenylephrine), and group E (Ephedrine). According to the randomization, hypotension was treated: with 100 μg Phenylephrine bolus IV, in Ph group or 5 mg bolus Ephedrine, in E group. If, at any time, maternal systolic blood pressure was < 80% baseline, the rescue doses of 100 μg Phenylephrine bolus IV, in Ph group or 5 mg bolus Ephedrine, in E group, were used. The doses of phenylephrine and ephedrine were chosen empirically, based on our clinical experience of the drugs. We recorded the number of total doses of vasopressors given up to the time of uterine incision. Heart rate and rhythm were monitored with ECG and any change from normal (tachycardia, bradycardia) were recorded and treated as needed. Bradycardia: HR < 60 bpm for 2 consecutive readings 1-minute apart [10]. If a patient developed bradycardia, 0.6 mg atropine was administered.

After delivery and clamping of umbilical cord, 1 mL blood was drawn from the umbilical artery for neonatal blood gas analysis.

The primary outcome was the incidence of maternal hypotension and secondary outcomes collected were: intra-operative maternal compli-cations (incidence of hypertension: SBP > 120% baseline, the incidence of bradycardia, the incidence of nausea: reported spontaneously by patients and vomiting: observed by investigators) and neonatal outcome parameters (the first- and fifth-minute Apgar scores: assessed by the attending paediatrician, and the umbilical artery blood gas analysis, obtained from a double-clamped segment of umbilical cord upon delivery). True fetal acidosis was defined as an umbilical arterial pH value of < 7.20 [14].

### Statistical analysis

Continuous variables were presented as the mean ± SD (standard deviation). Categorical variables were expressed as actual numbers (n) and percentages (%). Chi-square analysis was applied to compare frequencies between ­groups, and Student’s t-tests, one-way ANOVA or non-parametric tests when necessary were employed for quantitative variable analysis. Analysis was performed using the statistical software Statistics Package for Social Scientists (SPSS) version 15.0. Statistical significance was considered to be at the level of P ≤ 0.05. All tests were two-tailed.

## Results

Two hundred and two (202) pregnant women at term were entered in this study. Details of maternal demographic characteristics are summarized in Table 1.

**Table 1:** Maternal Characteristics

	Group E	Group Ph	P value
Age (year)	31.5 ± 5.34	30.26 ± 4.11	0.06
Weight (kg)	81.29 ± 12.66	79.74 ± 11.56	0.18
Height (cm)	165.49 ± 3.02	166.02 ± 5.47	0.39
Gestational age (weeks)	39.14 ± 0.85	39.07 ± 0.76	0.73
Induction-to-delivery time (min)	9.54 ± 2.35	9.47 ± 1.97	0.81

Data are given as mean ± SD.

The two groups were similar with respect to maternal age, weight, height, parity, and gestational age. Indications for cesarean section were repeated cesarean in 112 (55.44%) patients, other obstetrical indications for cesarean (cephalopelvic disproportion, breech or other abnormal presentations) in 77 (38.11%), and in 13 patients (6.43%) were patient’s preference. The time from induction of spinal anesthesia to delivery was similar in both groups (range from 5 to 15 minutes). Maternal hemodynamic variables are detailed in Table 2.

**Table 2:** Comparison of systolic blood pressure (BP) and heart rate (HR) between two groups

	Group E	Group Ph	P value
Basal Systolic BP (mmHg)	114.75 ± 8.46	116.72 ± 7.72	0.08
Systolic BP (mmHg) after anesthesia	82.0 ± 8.95	90.05 ± 6.40	< 0.0001
Systolic BP (mmHg) after vasopressor therapy	111.79 ± 15.96	118.13 ± 10.31	0.0009
HR at baseline (bpm)	101.78 ± 15.77	97.9 ± 17.16	0.09
HR after anesthesia (bpm)	68.81 ± 10.29	66.68 ± 8.23	0.10

Data are given as mean ± SD

### Maternal complications

**Figure 1:**
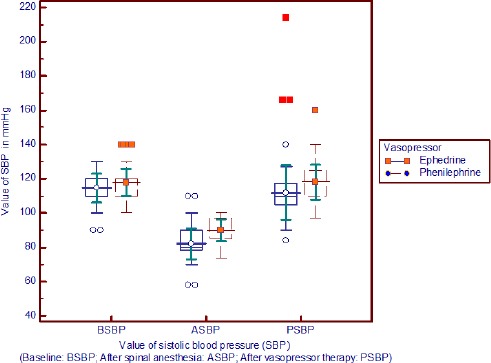
*Comparison of systolic blood pressure value between groups*.

Hypotension: 52 patients (51.48%) in group E and 56 patients (55.44%) in group Ph had persistent severe hypotension and needed additional vasopressor therapy. There was no significant difference between the groups regarding the number of doses of vasopressor required. (P = 0.82) (Figure 1).

In group E, 25 patients (24.75%) experienced bradycardia and needed one dose Atropine. 34 patients (23.76%), in group Ph had bradycardia. There was no significant difference between groups regarding the incidence of bradycardia (P = 0.65).

Nineteen (19) patients (16.83%) in group E and 12 patients (11.88%) in group Ph had nausea. Vomiting occurred in 4 patients (3.96%) in group E and in 10 patients (9.9%) in group Ph. There was no significant difference when comparing the incidence of nausea and vomiting between groups, respectively P = 0.87, and P = 0.47.

### Neonatal complications

First-minute Apgar scores were as follows: 6 neonates of group E (5.94%) and 7 neonates of group Ph (6.93%) had Apgar score 7; 17 neonates of group E (16.83%) and 12 neonates (11.88%) of group Ph had Apgar score 8; 78 neonates of group E (77.22%) and 82 neonates (81.18%) of group Ph, had Apgar score 9. There was no significant difference between groups regarding the first-minute Apgar scores.

Fifth-minutes Apgar scores were: two neonates in group E (1.98%) and none neonate (0.0%) in group Ph had mean Apgar score 8; 44 neonates (43.56%) in group E and 73 neonates (72.27%) in group Ph had mean Apgar score 9; 55 neonates in group E (54.45%) and 28 neonates (27.72%) in group Ph had a mean Apgar score of 10. Also there was no significant difference between groups regarding the fifth-minute Apgar scores.

At 1 and 5 min, no neonate in the Ephedrine or Phenylephrine groups had an Apgar score value of < 7.

**Figure 2:**
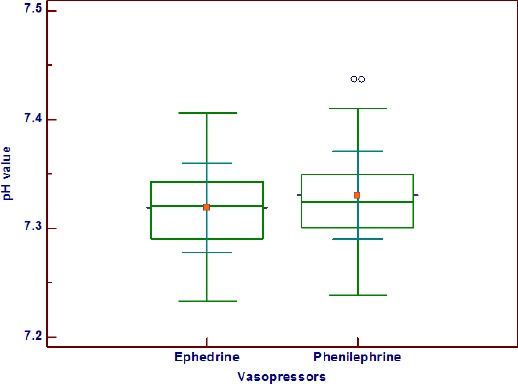
*Comparison of umbilical arterial blood pH value between the study groups*.

Umbilical arterial blood gas analyses are summarized in Table 3. Based on the one-way ANOVA test, there was a significant difference in PH between groups. Umbilical arterial PH was significantly lower in ephedrine group (P = 0.0005), but none of the neonates had the true fetal acidosis (Figure 2). There were no differences between groups regarding HCO_3_ concentrations and base excess values (Be) in the umbilical arterial blood gas analyses.

**Table 3:** Neonatal Data

	Total	Group E	Group Ph	P value
Umbilical arterial PH	7.32 ± 0.04	7.31 ± 0.04	7.33 ± 0.04	0.0005
HCO_3_ (mmHg)	22.55 ± 2.10	22.57 ± 1.87	22.52 ± 2.32	0.86
Base excess (mmol.l^-1^)	- 3.39 ± 1.90	- 3.48 ± 2.02	- 3.30 ± 1.78	0.50

Data are given as mean ± SD

## Discussion

In this study, we showed no significant differences between ephedrine and phenylephrine in their efficacy for treating spinal-induced intra-operative hypotension during cesarean sections. Also, there were no differences on maternal and neonatal outcomes, although parturients treated with phenylephrine had neonates with higher umbilical pH value than those treated with ephedrine. These results were consistent with the systematic review performed by Lee at al [8] and the meta-analysis performed by Lin et al [15].

As in other studies, [8, 10, 12, 16, 17] we showed there was no difference between groups for the treatment of maternal hypotension with Ephedrine and Phenylephrine. Lin et al [15] in a recent meta-analysis showed that when used to treat hypotension, patients given ephedrine and phenylephrine had comparable incidence of intra-operative hypotension. Our findings support the fact that ephedrine and phenylephrine are equally effective in controlling maternal hypotension [18].

In the contrary with other previous studies, [8, 10, 11, 19] that showed that patients in the Phenilephrine group were more likely than the Ephedrine group to develop bradycardia, in the present study there was no difference on the incidence of bradycardia between two groups.

Cooper showed that ephedrine use, compared with phenylephrine, was associated with a higher incidence of maternal nausea and vomiting [20]. We didn’t find any difference in the incidence of maternal nausea and vomiting between two groups.

The findings in the present study are in accordance with other studies [21, 22]: women given phenilephrine had neonates with higher umbilical arterial pH values than those given ephedrine, although there is no risk for true fetal acidosis in none of groups.

The reason for the difference in umbilical arterial pH values is that ephedrine crosses the placenta; [17, 22, 23] therefore, it is possible that ephedrine may have a direct effect on the fetus that contributes to acidosis [8]. In spite of this, fetal clinical adverse effects caused by reduced fetal pH have not been demonstrated [24].

In the present study none of the neonates had Apgar scores < 7, so there was no difference in the risk of low Apgar scores at 1 min or at 5 min (< 7) between the Ephedrine and Phenylephrine groups [8, 25].

### Limitations on the study

One possible limitation is that the study was conducted in a single center. Also, it was focused only in elective cesarean section and in healthy non-laboring women, not considering other conditions affecting maternal or neonatal outcome.

In conclusion, which of the two vasopressors: ephedrine or phenylephrine, is superior in treating spinal-induced intra-operative hypotension during cesarean sections, has been argued for years. In summary, this study supports the idea that ephedrine and phenylephrine have the same efficacy in treating hypotension after spinal anesthesia for caesarean section.

The use of Phenylephrine was associated with better fetal acid-base status compared to the use of ephedrine, but there were no differences on Apgar score values and on the incidence of the maternal bradycardia and hypotension after the use ofphenilephrine or ephedrine for treating spinal-induced intra-operative hypotension during cesarean sections.
